# Systemic vasculitis with latent tuberculosis infection and associated factors: a cross-sectional multicenter study

**DOI:** 10.1007/s10067-024-07279-7

**Published:** 2025-01-21

**Authors:** Jingjing Zhong, Yuanchun Li, Yan Chen, Xiaochun Shi, Baotong Zhou, Guiren Ruan, Lifan Zhang, Xiaoqing Liu, for the ETHERTB study team

**Affiliations:** 1 Division of Infectious Diseases, Department of Internal Medicine, State Key Laboratory of Complex Severe and Rare Disease, Peking Union Medical College Hospital, Chinese Academy of Medical Sciences & Peking Union Medical College, Beijing, 100730 China; 2 Clinical Epidemiology Unit, International Epidemiology Network, Chinese Academy of Medical Sciences & Peking Union Medical College, Beijing, 100730 China; 3 Center for Tuberculosis Research, Chinese Academy of Medical Sciences and Peking Union Medical College, Beijing, China

**Keywords:** Behçet’s syndrome, Latent tuberculosis infection, Systemic vasculitis, T-SPOT.TB

## Abstract

**Objectives:**

Systemic vasculitis patients are at a higher risk of developing latent tuberculosis infection (LTBI). However, there is currently no literature elucidating the positivity rate and risk factors for LTBI in systemic vasculitis patients.

**Methods:**

Our study is a multi-center, cross-sectional study that enrolled systemic vasculitis patients from 13 comprehensive hospitals in China. T-SPOT.TB as the screening method for LTBI, the study investigated the positivity rate of LTBI in systemic vasculitis patients and the factors associated with T-SPOT.TB results.

**Results:**

A total of 191 systemic vasculitis patients were included and the positive rate of T-SPOT.TB was 31.4%. The highest T-SPOT.TB positivity rate was observed in Behçet’s syndrome (BD) (72/191, 37.7%). There were statistically significant differences between the LTBI group and non-LTBI group in terms of systemic vasculitis type (*P* = 0.010), albumin levels (*P* = 0.034), erythrocyte sedimentation rate (*P* = 0.016), and corticosteroid dosage (*P* = 0.047). Multivariate regression analysis revealed that smoking history (aOR = 3.809, 95%CI: 1.341–10.817) and BD (aOR = 2.106, 95%CI: 1.042–4.254) were independent risk factors of T-SPOT.TB postive results, besides decreased lymphocyte count (aOR = 0.114, 95%CI: 0.013–0.973), and high-dose glucocorticoids use (aOR = 0.386, 95%CI: 0.149–1.003) were independent risk factors of T-SPOT.TB negative results.

**Conclusions:**

The prevalence of LTBI is high in systemic vasculitis patients, especially those with BD or smoking history. Patients with decreased lymphocyte counts and high-dose glucocorticoid use are more likely to have a negative T-SPOT.TB results. Therefore, LTBI screening should be performed based on the characteristics of the patient during the diagnosis and treatment of systemic vasculitis.

**Table:** 

**Key Points** • *We explored the positivity rate and risk factors of LTBI in systemic vasculitis patients from 13 hospitals in China.* • *There were 191 systemic vasculitis patients in our study. The positive rate of T-SPOT.TB was 31.4%. The predominant type of systemic vasculitis was BD, with a T-SPOT.TB positive rate of 44.4%. The second type was TA, with a T-SPOT.TB positive rate of 25.0%.* • *The prevalence of LTBI is high in systemic vasculitis patients, especially those with Behçet’s syndrome or smoking history. Decreased lymphocyte counts and high-dose glucocorticoid use are more likely to have a negative T-SPOT.TB results.* • *LTBI screening using T-SPOT.TB should be conducted during the diagnosis and treatment of systemic vasculitis.*

## Introduction

Tuberculosis is a kind of infectious disease caused by *Mycobacterium tuberculosis* (*Mtb*), which is a considerable disease burden worldwide [1]. Approximately one-third of the global population is estimated to be infected with either latent or active tuberculosis [2]. China is a country with a high burden of tuberculosis, and the population with latent tuberculosis infection (LTBI) is substantial [3]. Systemic vasculitis is a kind of autoimmune disease that primarily affects vessels (usually arteries or capillaries) [4]. Immunological disturbances associated with disease and immunosuppressive effects of disease-modifying antirheumatic drug (DMARDs) therapies may increase the risk of progression of LTBI to active tuberculosis (ATB) infection [5]. Therefore, early screening of LTBI and providing TB preventive therapy in patients with systemic vasculitis are particularly important.

There are two common screening tests for LTBI: tuberculin skin test (TST) and interferon-gamma release assays (IGRAs). IGRAs are recommended as first-line diagnostic tests for latent tuberculosis infection (LTBI) [6]. There are two commercially available IGRAs: a whole-blood IGRA (QuantiFERON-TB Gold In-Tube, QFT-GIT) and an enzyme-linked immunospot assay (T-SPOT.TB) [7]. Research has found that T-SPOT.TB has a higher positivity rate [8]. Neither of these methods can provide a definitive diagnosis of LTBI or distinguish ATB from LTBI [9]. If ATB is excluded by combining clinical presentation, imaging examination, etc., then LTBI can be diagnosed.

This study is based on a multicenter, cross-section study in China by our team, using T-SPOT.TB is the screening method for LTBI to investigate the LTBI infection status in rheumatic immune patients. The study results of LTBI have been published in Chinese [10]. However, there is currently limited basic research and clinical data on the combination of systemic vasculitis and LTBI. It is difficult for us to fully understand the prevalence of LTBI in patients with systemic vasculitis. Therefore we aim to explore the risk factors for LTBI in systemic vasculitis patients, providing a basis for early prevention and treatment of tuberculosis infection in patients with systemic vasculitis.

## Materials and methods

### Study design and patient recruitment

This study is a multicenter, cross-sectional research. It included rheumatic immune patients from 13 tertiary comprehensive hospitals in the eastern, central, and western regions of China from September 2014 to March 2016. All study subjects signed informed consent forms. Inclusion criteria: (1) Age over 15 years; (2) Diagnosed as Systemic vasculitis; (3) Completed T-SPOT.TB testing. Exclusion criteria: Clinical diagnosis or pathogen/tissue histopathological confirmation of ATB. This study was approved by the Ethics Committees of Peking Union Medical College Hospital (No. S-715) and 12 participating hospitals. Written informed consents were obtained from all patients and their legal guardians if necessary.

### Data collection

#### Clinical information

Collecting the basic information, including gender, age, medical history, previous tuberculosis history, and contact history. Collecting the clinical information, including disease type, duration, involvement of various systems, dose, and course of glucocorticoid (GC), immunosuppressants, and biological agents.

#### T-SPOT.TB assay

The T-SPOT.TB assay kit (Oxford Immunotec, UK) and the ELISPOT reader (Ispot Reader Spectrum, AID, Germany) were used in this study. A difference in spot counts between any antigen well and the negative control well ≥ 6, and at least 2 times the spot count of the negative control, was considered positive. If the spot count in the negative control well was > 10 or the spot count in the positive control well was < 20, the result was considered indeterminate.

### Statistical analysis

Data analyses were conducted using SPSS version 25 for Windows (IBM crop). For normally distributed metric data, descriptive statistics are reported as mean ± standard deviation, while for skewed distributed metric data, descriptive statistics are reported as median (interquartile range). Count data is described using percentages or composition ratios (%). *t*-tests and *Mann–Whitney* U tests were used for continuous data, and chi-square and Fisher exact tests were used for categorical data. Multivariable logistic regressions were used to identify the risk factors of LTBI. Variables that were considered clinically relevant, or variables which *P* value < 0.05 by chi-square were included in the multivariable logistic regression model (backward LR; entry, 0.05; removal, 0.10). *P* values < 0.05 were considered statistically significant.

## Results

### Prevalence of LTBI

A total of 191 systemic vasculitis patients who completed the T-SPOT.TB assay were included in this study. There were 60 cases of systemic vasculitis patients who tested positive for T-SPOT.TB, resulting in a positive rate of 31.4%. The predominant type of systemic vasculitis was BD (72/191, 37.7%), with a T-SPOT.TB positive rate of 44.4%. The second type was TA (36/191, 18.8%), with a T-SPOT.TB positive rate of 25.0%. There was no significant statistical difference in the T-SPOT.TB positive rate between different genders. Patients with systemic vasculitis aged 51 to 60 had the highest T-SPOT. TB-positive rate, which was 55.6% (Table 1).

**Table 1:** Prevalence of LTBI among systemic vasculitis patients of different disease types, genders, and age groups

	NO. of systemic vasculitis patients with LTBI	NO. of systemic vasculitis patients	Prevalence of LTBI (95%CI)	*P* value
Systemic vasculitis type				0.073
TA	9	36	25.0% (10.1%, 39.9%)	
GCA	2	4	50.0% (−41.9%, 141.9%)	
PAN	2	6	33.3% (−20.9%, 87.5%)	
GPA	7	38	18.4% (5.5%, 31.3%)	
MPA	6	29	20.7% (5.0%, 36.4%)	
EGPA	2	6	33.3% (−20.9%, 87.5%)	
BD	32	72	44.4% (32.7%, 56.2%)	
Gender				0.659
Male	30	91	33.0% (23.1%, 42.8%)	
Female	30	100	30.0% (20.9%, 39.1%)	
Age (yrs)				0.097
15 ~ 20	3	7	42.9% (−6.6%, 92.3%)	
21 ~ 30	10	42	23.8% (10.4%, 37.2%)	
31 ~ 40	10	34	29.4% (13.3%, 45.5%)	
41 ~ 50	12	36	33.3% (17.2%, 49.5%)	
51 ~ 60	15	27	55.6% (35.5%, 75.6%)	
61 ~ 70	8	36	22.2% (8.0%, 36.5%)	
> 70	2	9	22.2% (−11.7%, 56.1%)	
Total	60	191	31.4% (24.8%, 38.1%)	-

*TA* Takayasu arthritis, *GCA* giant cell arthritis, *PAN* polyarteritis nodosa, *GPA* granulomatosis with polyangiitis, *MPA* microscopic poly arteritis, *EGPA* eosinophilic granulomatosis with polyangiitis, *BD* Behcet’s disease

### Clinical characteristics

Patients were classified into LTBI (*n* = 60) and non-LTBI (*n* = 131) groups based on the results of T-SPOT.TB assay. The clinical characteristics of systemic vasculitis patients are shown in Table 2. The median age in the LTBI group was 49.5 (34.0, 58.0) years, which was higher than the median age of 42 (29.0, 61.0) years in the non-LTBI group. There were statistically significant differences between the LTBI and non-LTBI groups in terms of the types of systemic vasculitis (*P* = 0.010), with a higher prevalence of BD in the LTBI group. This study collected multiple laboratory parameters and identified statistically significant differences between the LTBI and non-LTBI groups in terms of albumin (*P* = 0.034) and erythrocyte sedimentation rate (ESR) (*P* = 0.016). The majority of patients received glucocorticoid therapy, with a dose of less than 60 mg/d being the most common (145/191, 75.9%). In the LTBI group, there were fewer patients receiving GC at a dose greater than 60 mg/d compared to the non-LTBI group. More than half of the patients were treated with immunosuppressants (114/191, 59.7%), predominantly using cyclophosphamide (79/114, 62.3%); biological agents were used less frequently (11/191, 0.6%). Fifty-six BD patients were treated with GC, and 10 of them had a dose of 60 mg/d or more. Thirty BD patients were treated with immunosuppressants, of which 20 were treated with cyclophosphamide, 2 with mycophenolate mofetil, 1 with methotrexate, 3 with azathioprine, 2 with leflunomide, 2 with cyclosporin A, and 3 with sulfasalazine. Three patients were treated with biological agents, including 1 with etanercept and 2 with infliximab.

**Table 2:** Clinical characteristics of systemic vasculitis patients with and without LTBI

	LTBI *n* = 60	non-LTBI *n* = 131	*P* value
Gender			0.659
Male	30 (50.0%)	61 (46.6%)	
Female	30 (50.0%)	70 (53.4%)	
Age (yrs)	49.5 (34.0, 58.0)	42 (29.0, 61.0)	0.581
BMI (Kg/m^2^)	22 (20.0, 24.0)	22 (20.0, 24.0)	0.732
Medical history			
Diabetes	3 (5.0%)	10 (7.6%)	0.502
Malignant tumor	1 (1.7%)	1 (0.8%)	0.569
Previous tuberculosis	6 (10.0%)	8 (6.1%)	0.338
Tuberculosis exposure history	3 (5.0%)	2 (1.5%)	0.163
Smoking history	11 (18.3%)	12 (9.1%)	0.071
Type of systemic vasculitis			0.010
TA	9 (15.0%)	27 (20.6%)	
BD	32 (53.3%)	40 (30.5%)	
Others	19 (31.7%)	64 (48.9%)	
Median course of systemic vasculitis (mo)	3 (1.0, 29.5)	4 (0, 31.0)	0.894
WBC (10^9^/L)			0.395
< 4	4 (6.7%)	5 (3.8%)	
≥ 4	56 (93.3%)	125 (95.4%)	
NEUT (10^9^/L)			0.925
< 1.5	2 (3.3%)	4 (3.1%)	
≥ 1.5	58 (96.7%)	126 (96.2%)	
LYM (10^9^/L)			0.065
< 0.5	2 (3.3%)	15 (11.5%)	
≥ 0.5	58 (96.7%)	115 (87.8%)	
HGB (g/L)			0.361
≥ 90	56 (93.3%)	115 (87.8%)	
< 90	4 (6.7%)	14 (10.7%)	
PLT (10^9^/L)			0.883
≥ 300	16 (26.7%)	36 (24.9%)	
< 300	44 (73.3%)	94 (71.8%)	
ALB (g/L)			0.034
≥ 30	54 (90.0%)	105 (80.2%)	
< 30	3 (5.0%)	21 (16.0%)	
ALT ( U/L)			0.755
Normal	53 (88.8%)	116 (88.5%)	
High	5 (8.3%)	13 (10.0%)	
BUN (mmol/L)			0.889
≤ 7.1	44 (73.3%)	96 (73.3%)	
> 7.1	14 (23.3%)	29 (22.1%)	
Cr			0.397
Normal	56 (93.3%)	117 (89.3%)	
High	3 (5.0%)	11 (8.4%)	
ESR			0.016
Normal	33 (55.0%)	50 (38.2%)	
High	24 (40.0%)	79 (60.3%)	
CRP			0.184
≤ 8	9 (15.0%)	29 (22.1%)	
> 8	14 (23.3%)	23 (17.6%)	
GC dose (mg/d)			0.047
< 60	51 (85.0%)	94 (71.8%)	
≥ 60	9 (15.0%)	37 (28.2%)	
Immunosuppressants, n/%	33 (55.0%)	82 (62.6%)	0.320
CTX	22 (36.7%)	57 (43.5%)	0.373
MMF	2 (3.3%)	6 (4.6%)	
MTX	3 (5.0%)	6 (4.6%)	0.899
AZA	2 (3.3%)	9 (6.9%)	0.330
LEF	1 (1.7%)	4 (3.1%)	0.577
CsA	1 (1.7%)	2 (1.5%)	0.942
FK506	1 (1.7%)	0	0.138
SASP	1 (1.7%)	2 (1.5%)	0.942
Tripterygium glycosides	0	4 (3.1%)	0.171
Biological agents, n/%	3 (5.0%)	8 (6.1%)	0.761
Etanercept	0	1 (0.8%)	0.138
Infliximab	2 (3.3%)	1 (0.8%)	0.185
Adalimumab	0	0	-
Tocilizumab	0	2 (1.5%)	0.336
Rituximab	0	5 (3.8%)	0.125

*TA* Takayasu arthritis, *BD* Behcet’s disease, *WBC* White blood cell, *LYM* Lymphocyte, *NEUT* Neutrophil, *HGB* Hemoglobin, *PLT* Platelet, *ALB* Albumin, *ALT* Alanine aminotransferase, *BUN* Blood urea nitrogen, *Cr* Creatinine, *ESR* Erythrocyte sedimentation rate, *CRP* C-reactive protein, *GC* glucocorticoid, *CTX* Cyclophosphamide, *MMF* Mycophenolate mofetil, *MTX* Methotrexate, *AZA* Azathioprine, *LEF* Leflunomide, *CsA* Cyclosporine A, *FK506* Tacrolimus, *SASP* Sulfasalazine

### Analysis of factors related to LTBI

Gender, age, previous tuberculosis, smoking history, lymphocyte count, albumin count, dose of GCs, the use of immunosuppressants and type of systemic vasculitis were included in a multivariable logistic regression analysis (Table 3). Smoking history (0R, 3.809; 95%CI, 1.314 to 10.817) and BD (0R, 2.106; 95%CI, 1.042 to 4.254) were identified as independent factors associated with positive T-SPOT.TB results. Lymphocyte count < 0.5*10^9^/L (0R, 0.114; 95%CI, 0.013 to 0.973) was identified as an independent factor associated with negative T-SPOT.TB results. Compared to non-smoking patients with systemic vasculitis, systemic vasculitis patients with a smoking history had a 3.809-fold increased risk of LTBI infection. Patients with BD are more prone to have LTBI. Patients with decreased lymphocyte count are more likely to have a negative T-SPOT.TB results. GC dose of ≥ 60mg/d is associated with negative T-SPOT.TB results.

**Table 3:** Multivariable logistic regression analysis of factors related to the results of T-SPOT.TB in systemic vasculitis patients

	No. of systemic vasculitis patients with positive T-SPOT.TB results	No. of systemic vasculitis patients with negative T-SPOT.TB results	aOR* (95%CI)
Smoking history	11 (90.8%)	12 (9.2%)	3.809 (1.341, 10.817)
LYM < 0.5*109/L	2 (3.3%)	15 (11.5%)	0.114 (0.013, 0.973)
ALB < 30g/L	3 (5.3%)	21 (16.7%)	0.276 (0.059, 1.296)
BD	32 (53.3%)	40 (30.5%)	2.106 (1.042, 4.254)
GCs does ≥ 60mg/d	9 (15.0%)	37 (28.2%)	0.386 (0.149, 1.003)

*LYM* Lymphocyte, *ALB* Albumin, *BD* Behcet’s disease, *GCs* GC

*aOR Adjusted odds ratios (OR) based on age and gender

## Discussion

China has one of the largest load of tuberculosis. According to the epidemiological survey in China in 2023, the estimated incidence of LTBI based on T-SPOT.TB was about 20%, and about 200 to 300 million people were infected. The management of LTBI is one of the important actions to realize the “End TB Strategy”. The risk of tuberculosis in patients with vasculitis treated with GCs and immunosuppressive is higher than that of the general population [11]. Patients have increased risk of LTBI before initiating disease-modifying antirheumatic drugs (DMARDs), immunosuppressants, and/or GCs. The risk for specific infections like TB differs in relation to dose and duration of treatment with GCs [12]. As recommended by EULAR in 2022, screening and management of chronic and opportunistic infections are very necessary [12]. IGRA used to screen for LTBI is an immunological-based test. Immunosuppressive therapy (GCs or immunosuppressive) in patients with vasculitis may affect IGRA results, which is a clinical concern. A study found that SLE patients treated with glucocorticoid doses of 60 mg/d or more (OR, 0.62; 95% CI, 0.39 to 0.98), leflunomide (LEF) (OR, 0.51; 95% CI, 0.29 to 0.88), or tacrolimus (FK506) (OR, 0.40; 95% CI, 0.16 to 1.00) were more likely to show negative results of T-SPOT.TB [13]. In the high-income country, the prevalence of active TB disease is 10/100,000, which has been decreasing over the past few decades. The load of LTBI has also remained small and steady [14]. Therefore, early detection and prevention of LTBI of systemic vasculitis patients in China are more crucial.

T-SPOT.TB was used in this study to assess LTBI. We found that the prevalence of LTBI among patients with systemic vasculitis was 31.4% (95% CI: 24.8% to 38.1%). The highest prevalence of LTBI was observed among patients with BD, reaching 44.4% (95% CI: 32.7% to 56.2%). Among patients with systemic vasculitis aged 51 to 60 years, the highest rate of LTBI was recorded at 55.6% (95% CI: 35.5% to 75.6%). A multicenter epidemiological study conducted in rural China found that the prevalence of LTBI among individuals aged 15 and above was approximately 20.3% (95% CI: 15.6% to 25.1%) and the prevalence of LTBI increased with age (*P* < 0.001) [15]. Patients with systemic vasculitis have a significantly higher prevalence of LTBI compared to the general population. Therefore, in the future diagnosis and treatment of systemic vasculitis, especially BD, more attention should be paid to the incidence of LTBI.

Our study found that smoking is an independent risk factor for LTBI. Among patients with systemic vasculitis, smokers have a significantly higher risk of LTBI compared to non-smokers, aligning with previous findings in non-rheumatic disease patients [16, 17]. However, the mechanisms of interactions between cigarette smoke and *Mtb* are unknown [18]. Some studies found that macrophage polarization is closely related to pulmonary tuberculosis and macrophage polarization will increase with smoking [19, 20], which may potentially suppress the immune response of the human body against TB. Many kinds of diseases will increase the risk of TB infection, such as HIV infection, diabetes mellitus, rheumatic diseases, silicosis, and chronic renal failure [21]. Population with a history of previous TB and exposure to tuberculosis are more likely to have a positive T-SPOT.TB test results [21, 22]. We did not observe significant statistical differences in our study, which may be attributed to the relatively small number of patients enrolled.

Systemic vasculitis is currently categorized based on the size of affected blood vessels according to the Chapel Hill classification, including Takayasu arteritis (TAK), giant cell arteritis (GCA), polyarteritis nodosa (PAN), ANCA-associated vasculitis (AAV), Behcet’s disease (BD), and so on [23]. A 2022 study found that vasculitis may occur as a secondary condition due to medications, infections, or other rheumatic diseases, with 31.9% of cases being secondary to tuberculosis (36/113). As a result, tuberculosis screening is essential in the diagnosis and management of patients with vasculitis [24]. BD is one of the most common types of systemic vasculitis. This study found that BD is the most common type of systemic vasculitis in inland China. Clinical research data suggests that BD is an independent risk factor for TB [25]. Epidemiological data from other population groups indicate that BD patients have a higher incidence of TB compared to non-BD patients [26]. Studies conducted in China found that the prevalence of LTBI among patients with BD was 29.3%, which is consistent with the data obtained in our study [27]. BD may produce a defect in cell-mediated immunity and BD patients are more susceptible to tuberculosis due to immunity defects [25, 28]. Anti-TNF-αagents for BD treatment weaken the immune response, which may increase the risk of developing TB [29].

Several factors can influence the result of T-SPOT.TB. Our research has found that decreased lymphocyte count and high-dose GC use are associated with negative T-SPOT.TB results. A study found that patients with a lymphocyte count of less than 500 cells/mm3 were more likely to have negative T-SPOT.TB results [30], which are consistent with our study. T-SPOT.TB provided more determinate results and was less influenced by lymphocytopenia than QFT-GIT [31]. In these special conditions, T-SPOT.TB is the preferred choice for TB testing. Although the statistical significance of high-dose glucocorticoid use in our study was borderline (*P* = 0.051), we still believe that the use of high-dose GC is an independent factor to negative T-SPOT.TB results. GC can suppress the human immune function in various ways [32]. For example, GC can induce lymphopenia across all lymphocyte subpopulations and inhibit T-cell activation by suppressing interleukins 2, 3, 4, and 6 [33]. It could be one of the potential reasons for the negative T-SPOT.TB results. GC can increase the risk of LTBI in rheumatic disease patients [34]. Other factors are also associated with the negative results of T-SPOT.TB, leading to uncertainty in the detection, such as advanced age, malnutrition, low CD4 + T-cell levels, and the use of immunosuppressive agents [30, 35].

## Limitations

This study has limitations. Firstly, our study is based on a previous multicenter, cross-sectional study. The target population was rheumatic autoimmune patients undergoing T-SPOT.TB testing may introduce selection bias. Secondly, the majority of patients in our study had BD, which could potentially affect the analysis of factors associated with T-SPOT.TB results.

## Conclusions

In the population of systemic vasculitis, LTBI is significantly more prevalent, with BD patients and smokers being at the highest risk. When systemic vasculitis patients use high-dose GC or have decreased peripheral blood lymphocyte counts, they are more likely to have negative T-SPOT.TB results. Therefore, during the diagnosis and treatment process of systemic vasculitis patients, T-SPOT.TB testing should be improved to screen for LTBI, and preventive anti-TB treatment should be implemented based on the patient’s situation. If patients have significantly decreased peripheral blood lymphocyte counts, even if T-SPOT.TB results are negative, and LTBI cannot be completely excluded. Close follow-up and vigilance for ATB should be maintained throughout the diagnosis and treatment process.

## Data Availability

All data are available from the corresponding author.
